# Supplementary material for: Entrainment dominates the interaction of microalgae with micron-sized objects
Source: Nat Commun. 2016 Aug 18;7:12518. doi: 10.1038/ncomms12518 (PMC4992151; doi:10.1038/ncomms12518)
Supplement: Supplementary Information — Supplementary Figure 1-9, Supplementary Notes 1-5, Supplementary Methods and Supplementary References [file ncomms12518-s1.pdf]

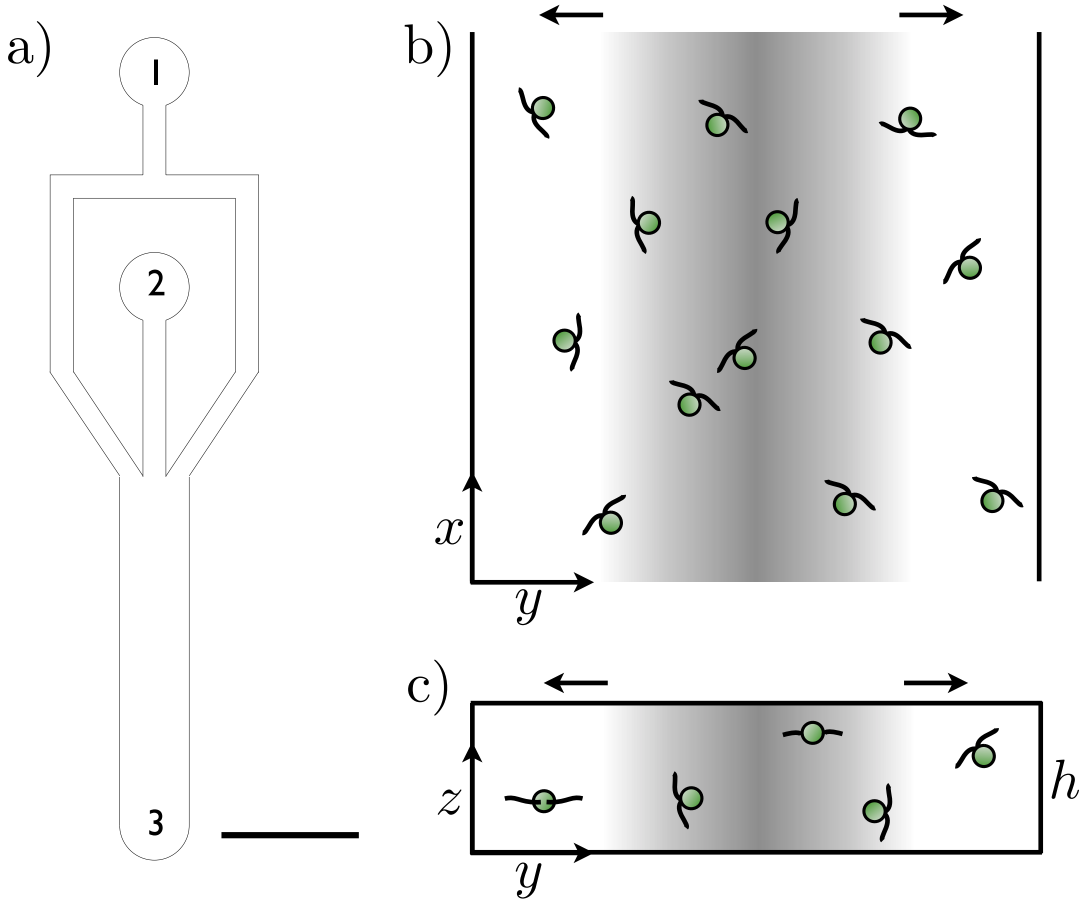


SUPPLEMENTARY FIGURE 1: a) Sketch of the microfluidic devices used for the spreading experiment. Inlet 1: suspension of CR only; inlet 2 suspension of CR and beads; 3 is the outlet. Scale bar: 4 mm. b) Sketch of the experimental configuration, top-view. The shaded area represents the band of colloid spreading along the width of the channel in presence of CR (green disks) c) Sketch of the experimental configuration, side-view. The shaded area represents the band of colloid spreading along the width of the channel. Height of the channel: $h = 60 \pm 5\ \mu$m.

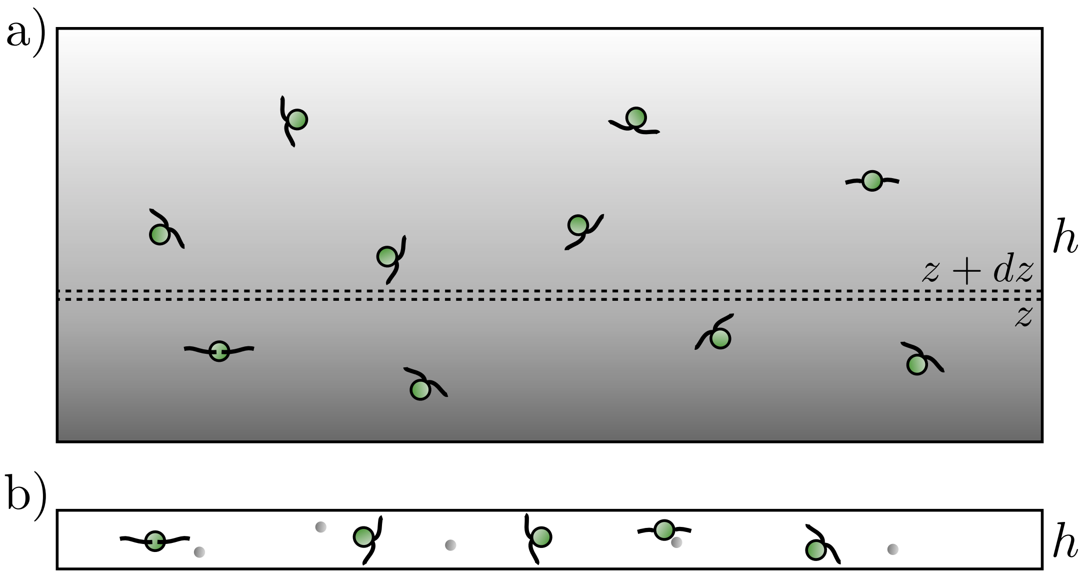


SUPPLEMENTARY FIGURE 2: a) Sketch of the experimental setup for the sedimentation experiment, side view. The suspension of CR and tracers is loaded into a 2 mm wide and $h = 185 \pm 20$ $\mu$m thick microfluidic device. Beads are then imaged in layers $[z, z + dz]$ about 1 $\mu$m thick by steps of 1 $\mu$m in order to reconstruct the density profiles. The shaded area represents the colloidal distribution. b) Sketch of the experimental setup for the Hele-Shaw experiment, side view. The suspension of CR and tracers is loaded into a 2 mm wide and $h = 25.8 \pm 0.1$ $\mu$m thick microfluidic device. The density of beads is kept very low in order to have only a few particles in the field of view and facilitate long-time tracking.

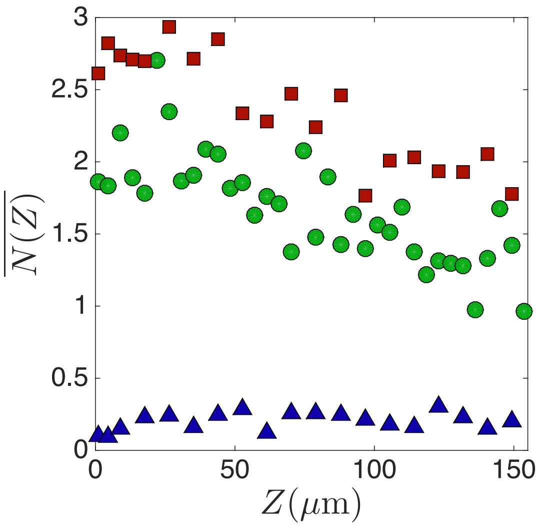


SUPPLEMENTARY FIGURE 3: Density profiles of algae at 3 different concentrations in the chamber shown in Supplementary Fig. 2a used for the sedimentation experiment. $N(Z)$ represents the time-averaged number of CR at altitude $Z$. Red squares: $N_c = 3.65 \pm 0.20 \times 10^6$cells/ml; Green circles: $N_c = 1.84 \pm 0.05 \times 10^6$cells/ml; Blue triangles: $N_c = 0.56 \pm 0.08 \times 10^6$cells/ml. The density is seen to decrease with the altitude, with a factor between 1.5 and 2 between the bottom and the top of the sample cell, except for the lowest concentration (blue triangles).

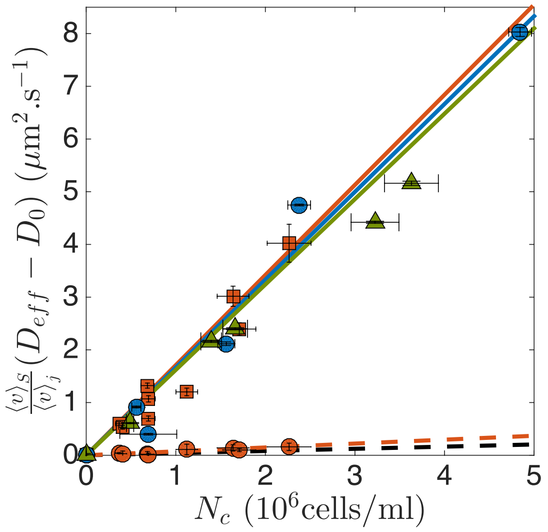


SUPPLEMENTARY FIGURE 4: Close up on low concentration values of Fig. 1c: Effective microparticle diffusivity $(D_{eff} - D_0)$ as a function of $N_c$ for the three experiments. Diffusivities are rescaled by the ratio of the average CR speeds $\langle v\rangle_S$ / $\langle v\rangle_j$ where $j$ stands for sedimentation ($S$), spreading ($CS$) or tracking ($HS$) experiments. Solid lines are best linear fits to the data. Orange squares: sedimentation experiment (slope $\alpha_S = 1.71 \pm 0.14$ ($\mu$m$^2$/s)/($10^6$ cells/ml); all other values in the same units); green triangles: spreading experiment ($\alpha_{CS} = 1.62 \pm 0.14$); blue circles: Hele-Shaw experiment ($\alpha_{HS} = 1.67 \pm 0.13$). Orange circles and dashed line: direct tracking in the sedimentation experiment ($\alpha_T = 0.074 \pm 0.014$). Black dashed line: fit to the experimental diffusivity obtained by direct tracking in [3] ($\alpha_L = 0.041$)

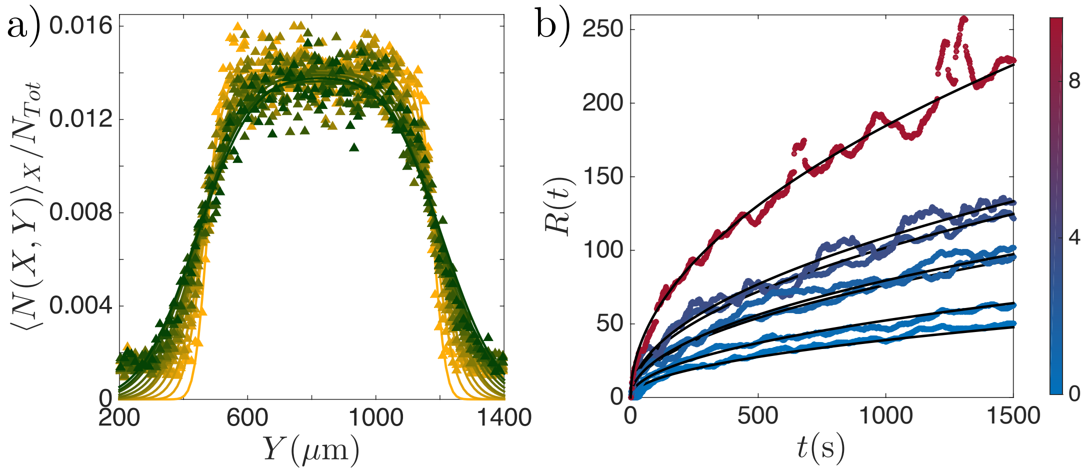


SUPPLEMENTARY FIGURE 5: Density profiles in the macroscopic spreading experiments. a) Time evolution of the density profile (averaged over the x-direction) of the band of colloids in the spreading experiment at $N_c = 9.63 \pm 0.80 \times 10^6$cells/ml. The colors code for the time, from the initial condition (yellow curve) to $t = 1050$s (dark green curve). The profiles are shown every 150s. The curves are fitted with the function: $\rho(y,t) = \rho_0/2[\text{erf}((y-y_0)/R(t)) - \text{erf}((y-y_0-\Delta y)/R(t))]$. b) Plot of the parameter $R(t)$ as a function of time obtained from the fits in (a) for the different algae concentrations used (the colors code for $N_c$, units: $10^6$cells/ml). $R(t)$ is supposed to evolve as $\sqrt{4D_{eff}t}$, which is exactly what we obtained. Black curves are best fits of this type, from which we extracted the effective diffusivity.

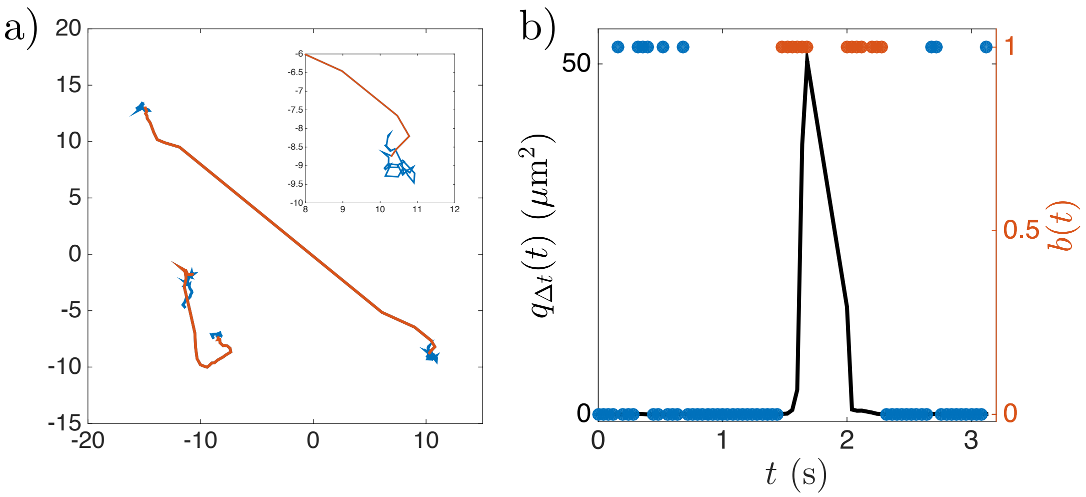


SUPPLEMENTARY FIGURE 6: Recognising jumps in microparticle trajectories. a) Typical pieces of trajectories isolated after the first step of the protocol (blue+orange curves). The central curve is due to particle entrainment by CR, while the lowest curve corresponds to a far-field perturbation by CR (loop-like trajectory). The orange parts of these curves correspond to the jumps (or loops) after removing the Brownian parts at the beginning and at the end. Inset: close-up on the end of the central curve showing that after the first step of the protocol, jumps (or loops) have been isolated together with thermal motion that needs to be removed. b) Black curve: evolution of $q_{\Delta t}(t)$ (see Supplementary Methods 3 for definitions of these quantities) for the central curve in (a). Blue and orange circles: evolution of b(t) for the central curve in (a). The orange circles correspond to the orange part of the trajectory in (a).

a) 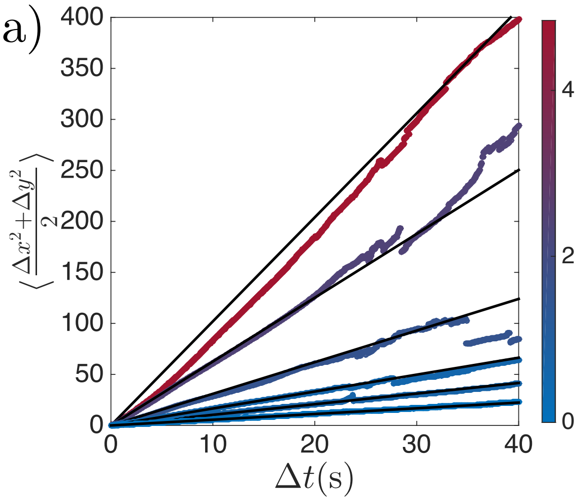


b)

TABLE I: Statistics of recorded trajectories

| Concentration ($10^6$cells/ml) | Number of recorded trajectories | Average duration (s) |
|---|---|---|
| 0 | 55 | 271 |
| 0.56 | 66 | 203 |
| 0.69 | 89 | 175 |
| 1.56 | 100 | 144 |
| 2.38 | 92 | 71 |
| 4.84 | 63 | 135 |

SUPPLEMENTARY FIGURE 7: Mean Square Displacement of the colloids in the Hele-Shaw experiment for the different algae concentrations (the colors code for $N_c$, units: $10^6$cells/ml). The MSD is seen to increase linearly with time delay in the long-time limit, showing that the jumps conserve the diffusive behaviour of the colloidal dynamics. Black lines are best linear fits to the data. These fits, needed to extract the effective diffusivity, have been performed on a range of time where the MSD is linear and non-noisy, which depends on the concentration. For the sake of clarity we show only the 40 first seconds, but some fits have been performed on longer time delays (for instance at the highest concentration, red curve).

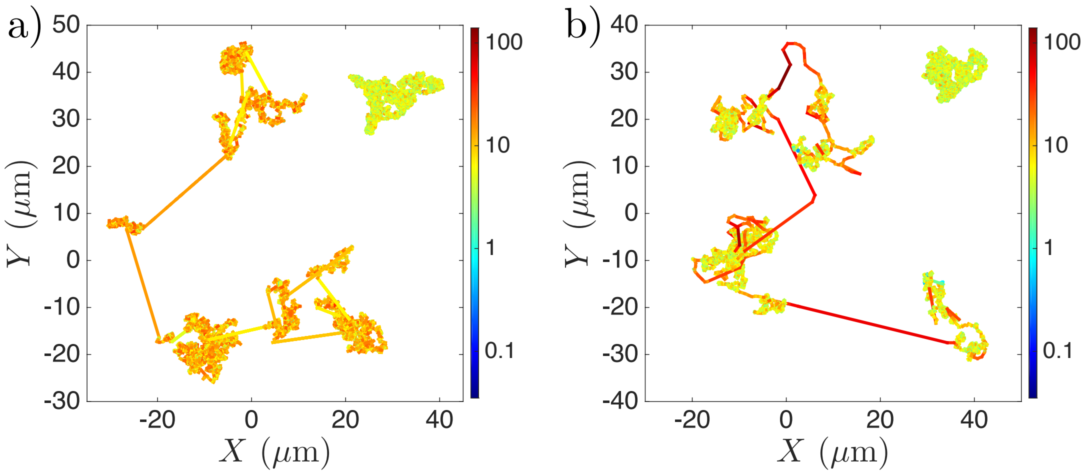


SUPPLEMENTARY FIGURE 8: Typical microparticle trajectories in simulations and experiments. (a) Typical trajectories ob-tained by numerical simulations at $N_c = 5 \times 10^6$cells/ml (center) and $N_c = 0$ (top-right). (b) Typical trajectories obtained in the experiments at $N_c = (4.84 \pm 0.13) \times 10^6$cells/ml (center) and $N_c = 0$ (top-right). The color code for the velocity, units: $\mu$m.s$^{-1}$. The 4 trajectories are $\sim$ 210s long and the time interval between 2 consecutive points is 0.04s in all cases. We readily see that the 2 trajectories for $N_c > 0$ are qualitatively very similar, but we observe a discrepancy on the velocities (see Supplementary Note 4). At equilibrium however, the numerical and experimental trajectories are indistinguishable.

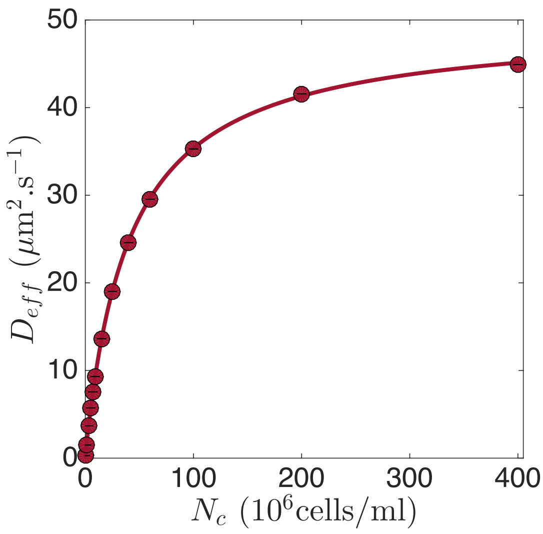


SUPPLEMENTARY FIGURE 9: Evolution of the effective diffusivity $D_{eff}$ in the simulations together with the fit performed using the theoretical formula leaving $u$ and $\langle L \rangle$ as free parameters. The agreement with the experimental values is reasonable (see Supplementary Note 5).

## SUPPLEMENTARY NOTE 1 : DENSITY PROFILE OF ALGAE IN THE SEDIMENTATION EXPERIMENT

The steady state particle profiles are set by the effective diffusivity, which depends on the concentration of CR. A non-uniform concentration of CR along the vertical direction would then lead in principle to a different steady state profile of beads, as the particles' effective diffusivity would depends on the altitude. We measured the concentration profile of CR in the device used for the sedimentation experiment at different average CR concentrations, see Figure 3. The local algal concentration is measured following the same procedure than the one used to obtain the colloids' profiles. Stacks of 1000 images were recorded at 10 fps at different altitudes in the sample cell in order to obtain the time-average number of CR $\overline{N(Z)}$ in each layer. These measurements have been done without colloids in the system, assuming that their presence would not alter the results. We found that the concentration tends to decrease with altitude, except for the smallest concentration (Supplementary Fig. 3, blue triangles). The low-concentration profile might differ due to subsampling, as for small concentration taking only 1000 images over 100 s might be not enough to evaluate accurately the time-averaged number of cells. Based on the two highest concentrations, we observe that the concentration at the top of the device is between 1.5 and 2 times smaller than the one at the bottom. We expect that this effect will lead to a gravitational length $l_{g,eff}$ higher than the one given by the effective diffusivity in a uniform active bath, because in that case the beads at the bottom diffuse more than those above. As a result, our measurement of the average effective diffusivity through the sedimentation experiment is probably slightly overestimated. The slope $\alpha_S$ is indeed the largest of the three experimental values obtained, but the difference with the other two is less than 4% ($2\alpha_S/(\alpha_{CS} + \alpha_{HS}) \simeq 1.04$). Consequently we do not consider the non-uniformity in CR concentration to affect the results appreciably, although it is possible that this effect will become important for other strains or species with a stronger bias.

## SUPPLEMENTARY NOTE 2 : PDF OF DISPLACEMENTS IN THE HELE-SHAW EXPERIMENTS

We detail here our results on the full statistics of colloids displacements. To better understand what is the consequence of the entrainment on the statistics of displacements, we have computed the PDF of displacements as a function of the time interval $\Delta t$, $PDF_{\Delta t}(\Delta x)$, when considering the jumps (blue curves in Fig. 4a and b) or after removing them from the trajectories (red curves in Fig. 4a and b). The curves presented Fig. 4 correspond to a fixed cell concentration $N_c = (4.84 \pm 0.13) \times 10^6$ cells/ml. The distributions with and without jumps, identical at very short $\Delta t$, differ more and more as the time interval increases. This a consequence of the increasing importance of jumps: the variance grows faster when the jumps are considered, which leads to much higher diffusivities. Both distributions

present large exponential-like tails at short and intermediate times. These tails reflect the effect on tracers' dynamics of loop-like perturbations alone (red curves) and with entrainments (blue curves). As $\Delta t$ increases, both distributions start to converge towards Gaussians, as predicted in [5] and in agreement with the Central Limit Theorem. This is confirmed by looking at the modified kurtosis, $\kappa(\Delta t) = \mu_4/\mu_2^2 - 3$ ($\mu_n$ being the n$^{th}$-centered moment of $PDF_{\Delta t}(\Delta x)$), which is a measure of deviation from Gaussian behaviour ($\kappa = 0$ for a Gaussian). Figure 4c and d show the evolution of $\kappa(\Delta t)$ at different cell concentrations for the distributions with and without jumps respectively (colorbar: $N_c$ in units of $10^6$ cells/ml). We see that $\kappa(\Delta t)$ converges to 0 in all cases, but the convergence is slower for the full distribution (i.e. with jumps). This is due to a combination of the large size of jumps and their low frequency. The distribution is expected to converge to a Gaussian only at $\Delta t$ large compared to the typical inter-jump waiting time. In fact, Fig. 4c shows that the convergence is faster as the cell concentration $N_c$ increases. The bottom curves (light blue) in Fig. 4c and d correspond to the equilibrium case (without CR) showing a Gaussian dynamics at all time as expected for Brownian particles.

## SUPPLEMENTARY NOTE 3 : MSD OF THE PARTICLES IN THE HELE-SHAW EXPERIMENT

From the particles trajectories recorder in the Hele-Shaw experiment, we measured their MSD for each algae's concentration used and deduced their effective diffusivity. We recorded an average of 77.5 trajectories per experiment lasting on average 166.5s. A more detailed table can be found in Supplementary Fig. 7b, where we show the number and the length of the recorded trajectories at each concentration. The measured MSD $\frac{1}{2}\langle(\Delta x^2 + \Delta y^2)\rangle$ is presented Supplementary Fig. 7a for $\Delta t$ up to 40s. We readily see that the long-time limit is linear, showing that the whole process (jumps entrainment+far-field contribution) keeps a diffusive nature: $\frac{1}{2}\langle(\Delta x^2 + \Delta y^2)\rangle = 2D_{\text{eff}}\,\Delta t$. The prefactor of 1/2 comes of course from the standard definition $\langle \Delta r^2\rangle_d = 2dDt$, with $d = 2$ for the Hele-Shaw experiments. The linear fits to obtain the effective diffusivity have been done on a range of $\Delta t$ where the MSD is consistently linear. For instance above $\Delta t = 25$s some of the curves are quite noisy, so we discarded this part in the fit (except for the highest concentration, red curve, where the fit has been done up to $\Delta t \sim 50$s).

## SUPPLEMENTARY NOTE 4 : TYPICAL TRAJECTORY FROM THE SIMULATIONS

As shown Supplementaty Fig. 8, the simulated trajectories are qualitatively very similar to the experimental ones. However we can notice that the velocities do not well agree. Indeed during the jumps, we observe that the velocity is smaller in the simulated trajectories. This can be explained easily by looking more closely at the experimental trajectory: the jumps are quite often curved due to the change of direction of CR during the entrainment process, but the simulation is based on straight jumps with a length given by the end-to-end length $L$ of the experimental jumps while the duration is the same. The jump velocity obtained in the simulation is then naturally smaller. Moreover looking at both trajectories we observe that the velocities in the blobs seem much more uniform in the simulation than in the experiment (the color is orangish in the simulation, while it goes mainly from green to red in the experiment). We can also provide a rational explanation for this. The dynamics in these blobs (for the experiment) is composed of 2 processes: a thermal Brownian motion characterized by the equilibrium diffusivity $D_0$ and a far-field perturbation due to the swimming of CR (the mechanism probed in [3]). This is clearly visible when looking closely at the velocities in these blobs and comparing them to the velocities obtained at equilibrium (top-right trajectory in Figure 8(b)). Regarding the simulation, in order to stay on the simplest level while keeping the essential ingredients relevant to our study, we decided to consider this 2-fold process (Brownian motion+far-field perturbation) as an effective Brownian motion with diffusivity $D_{WJ}$. This naturally leads to the observed discrepancy on the velocities. This difference is of course removed when setting the concentration of cells $N_c$ to 0 in the simulation, see Supplementary Fig. 8a top-right trajectory. This trajectory is indeed indistinguishable from the experimental one.

## SUPPLEMENTARY NOTE 5 : ANALYTICAL STUDY OF A JUMP-DIFFUSION PROCESS

In this section, we develop an analytical mesoscopic theory to account for the evolution of the effective diffusivity with the concentration of algae. This theory is a 2D version of the 1D diffusion-ballistic process developed in [4].

We consider 2 population densities $\rho_d(x, y, t)$ and $\rho_b(x, y, t, \phi)$, corresponding respectively to particles diffusing (with diffusivity $D_{WJ}$) at position $(x, y)$ at time $t$ and to particles at position $(x, y)$ at time $t$ moving ballistically with a constant velocity $v$ in the direction $\phi$. The switching rate from diffusing behavior to ballistic motion (in any

direction) is called $\lambda_d$, while the switching rate from ballistic motion to diffusion is called $\lambda_b$. The total density is then $\rho(x,y,t) = \rho_d(x,y,t) + \int_0^{2\pi} \rho_b(x,y,t,\phi)d\phi$. Finally, we consider that the whole population is initially centered at the origin. The equations of evolution of the densities are:

$$\begin{aligned} \frac{\partial \rho_d}{\partial t} &= D_{WJ}\Delta\rho_d - \lambda_d\rho_d + \lambda_b \int_0^{2\pi} \rho_b d\phi \\ \frac{\partial \rho_b}{\partial t} &= -u\cos(\phi)\frac{\partial \rho_b}{\partial x} - u\sin(\phi)\frac{\partial \rho_b}{\partial y} + \frac{\lambda_d}{2\pi}\rho_d - \lambda_b\rho_b \end{aligned} \tag{1}$$

We then Fourier-Laplace transform this set of equations which leads to:

$$\begin{aligned} s\hat{\rho}_d - \rho_d(0) &= -(k_x^2 + k_y^2)D_{WJ}\hat{\rho}_d - \lambda_d\hat{\rho}_d + \lambda_b \int_0^{2\pi} \hat{\rho}_b d\phi \\ s\hat{\rho}_b - \rho_b(0) &= -ik_x u\cos(\phi)\hat{\rho}_b - ik_y u\sin(\phi)\hat{\rho}_b + \frac{\lambda_d}{2\pi}\hat{\rho}_d - \lambda_b\hat{\rho}_b \end{aligned} \tag{2}$$

From which we can easily (but tediously) get the total density $\hat{\rho} = \hat{\rho}_d + \int_0^{2\pi} \hat{\rho}_b d\phi$, that we will not reproduce here for the sake of clarity. $\rho_d(0)$ and $\rho_b(0)$ represent here respectively the fraction of diffusing particle and the fraction of ballistic particles (moving in the direction $\phi$) at time $t = 0$ (and being at the origin). We then have $\rho(0) = \rho_d(0) + 2\pi\rho_b(0) = 1$. The first and second moment $\langle x\rangle$ and $\langle x^2\rangle$ are given by the formula :

$$\langle x\rangle = i \lim_{\substack{k_x\to 0\\ k_y\to 0}} \frac{\partial \hat{\rho}}{\partial k_x}\ , \qquad \langle x^2\rangle = -\lim_{\substack{k_x\to 0\\ k_y\to 0}} \frac{\partial^2 \hat{\rho}}{\partial k_x^2} \tag{3}$$

We obtain:

$$\langle x\rangle = 0\ , \qquad \langle x^2\rangle = \frac{2D_{WJ}(\lambda_b + s)(\lambda_b\rho(0) + \rho_d(0)s) + (\lambda_d\rho(0) + 2\pi\rho_b(0)s)u^2}{s^2(\lambda_b + s)(\lambda_d + \lambda_b + s)} \tag{4}$$

By inverse Laplace transforming we then obtain the evolution of the second moment in time. The formula is quite lengthy so we do not reproduce it here, but we can easily write the asymptotic behavior:

$$\begin{aligned} \lim_{t\to 0}\langle x^2\rangle &= 2D_{WJ}\rho_d(0)t \\ \lim_{t\to\infty}\langle x^2\rangle &= 2\left(\frac{D_{WJ}\lambda_b}{\lambda_d + \lambda_b} + \frac{\lambda_d u^2}{2\lambda_b(\lambda_d + \lambda_b)}\right)\rho(0)t \end{aligned} \tag{5}$$

Finally from this asymptotic behavior, we see that the effective diffusivity associated to this process is:

$$D_{eff} = \frac{D_{WJ}\lambda_b}{\lambda_d + \lambda_b} + \frac{\lambda_d u^2}{2\lambda_b(\lambda_d + \lambda_b)} \tag{6}$$

We can now try to relate this theory with our experiment/simulation. We consider that the diffusivity $D_{WJ}$ is the diffusivity when the jumps are removed from the trajectories, $D_{WJ} = \alpha_{WJ}N_{cells} + D_0$ with $\alpha_{WJ} = 0.20 \pm 0.03$ and $D_0 = 0.28 \pm 0.02\mu\text{m}^2.\text{s}^{-1}$. The switching rate from diffusion to ballistic is given by $\lambda_d = 1/\langle\Delta T_J\rangle = \gamma v N_c$ (with $v = \langle v\rangle_{HS} = 49.1\mu\text{m.s}^{-1}$ and $\gamma = 299 \pm 35\,\text{mm}^2$). The switching rate from ballistic motion to diffusion is given by $\lambda_b = \frac{u}{\langle L\rangle}$. Altogether this leads to:

$$D_{eff} = \frac{D_0 + \alpha_{WJ}N_c + \frac{\langle L\rangle^2}{2}\gamma v N_c}{1 + \frac{\langle L\rangle}{u}\gamma v N_c} \tag{7}$$

This theory account for a process in which the ballistic motion is characterized by a constant velocity $v$ and a switching rate $\lambda_b$ between ballistic and diffusive behavior. In the simulations however, we set the duration of the jumps as being constant equal to $\tau = 1.7$s and with a length $L$ taken in the distribution $PDF(L)$ in order to be the most faithful to the experiments. The processes are then slightly different. Nonetheless we can use this formula to fit the evolution of $D_{eff}(N_c)$ in the simulation, leaving $\langle L\rangle$ and $u$ as free parameters and taking the other parameters from the experimental values. The fit is very good as shown in Supplementary Fig. 9. The jump velocity obtained from this fit is $u = 7.07 \pm 0.08\mu\text{m.s}^{-1}$, which is $\sim 39\%$ smaller than the average jump velocity measured in the experiments ($11.6\mu\text{m.s}^{-1}$). This experimental value has been measured by looking at the distribution of jumps velocities defined by the end-to-end length of jumps divided by the duration of the jumps. Regarding the average jumps length, the fit gives $\langle L\rangle = 11.74 \pm 0.12\mu\text{m}$, which is $\sim 22\%$ smaller than the experimental value ($15\mu\text{m}$).

# SUPPLEMENTARY METHODS

## SUPPLEMENTARY METHODS 1 : MICROFLUIDIC DEVICES

Microfluidic channels were cast in PDMS from SU8 master mold, which was produced using standard soft lithography techniques. The device for the sedimentation experiment was a channel 2 mm wide and $185 \pm 20\,\mu$m thick. For the Hele-Shaw tracking experiment, we used a rectangular channel 2 mm wide and $25.8 \pm 0.1\,\mu$m thick. Finally the spreading experiments were performed in a 3-arms fork-shape channel 2 mm wide and $60 \pm 5\,\mu$m thick, see Supplementary Fig. 1a.

## SUPPLEMENTARY METHODS 2 : SPREADING EXPERIMENTS

The band of colloids for the spreading experiment was initiated by injecting the CR+beads Percoll solution (solution 1) in the inlet 2 and the CR Percoll solution (solution 2) only in the inlet 1, see Supplementary Fig. 1a. To do so, a $100\mu$l gas-tight syringe was filled with solution 1 and a $50\mu$l gas-tight syringe was filled with solution 2. The 2 syringes were then placed on a PHD 2000 Harvard Apparatus syringe pump and connected to inlets A and B. Applying the same flow-rate to the 2 syringes ensure that the flow rate is the same in the 3 branches connecting the main channel. This procedure allows then to obtain a sharp density profile with all the beads uniformly distributed in the central region and no bead in the sided regions, while CR are uniformly distributed in the whole sample. We started to record the dynamics right after stoping the flow and closing the outlet with a tap in order to prevent flows due to the evaporation of the solution.

Also we should explain why we used a device having a thickness of $60\mu$m for this experiment. The experiment consists in studying the diffusive spreading of a band of colloids in bulk. In order to remove any kind of convection such as gravity currents, we density-matched the liquid with the colloids. Indeed gravity current is a phenomenon appearing as soon as a heavy fluid has a vertical interface with a lighter fluid and consists in the horizontal displacement of the heavy fluid into the lighter fluid at the bottom of the system. This problem is a very complicated one but the origin of the instability is easily understandable: the hydrostatic pressure at the bottom of the system from both side of the interface is not the same, it is higher in the heavier fluid. This mismatch of pressure leads to a flow originated at the bottom of the system from the heavy fluid to the lighter fluid. Due to mass conservation, the lighter fluid flows also into the heavy fluid at the top of the system (in a close container). This instability has no threshold, as the pressure difference will be always non-zero as soon as the 2 fluids has slightly different densities. While doing the first experiments in a $400\mu$m thick channel, we were observing these gravity currents which were preventing us from studying the diffusing spreading. Indeed the density-matching procedure is never perfect and as a consequence we had a mismatch of density between the band and the rest of the system. If we cannot remove completely this phenomenon, we can still increase the time scale associated with it in order to have the initial dynamics dominated by diffusion. And if we cannot easily improve the density matching procedure, we can reduce the hydrostatic pressure at the bottom of the system by reducing the thickness of the device in order to increase sufficiently the time scale of this convection. We verified that taking a $60\mu$m-thick channel was enough to reduce this effect and have a dynamics dominated by diffusion.

We now explain how the analysis of the spreading experiment has been done. As mentioned previously, the experiment is done by taking images of the system every 2s for several hours with an SLR camera. The spatial resolution of the pictures is big enough to localize the beads. The analysis consists in computing the density profile along the spreading direction at each time step in order to study its evolution and deduce the effective diffusivity of the particles. We will first explain how we obtained the density profiles and then how we got the effective diffusivity from the spreading dynamics.

We obtained the density profile along the spreading direction by binning it in 142 bins (corresponding to a width of $\sim 11\mu$m) and by averaging over the perpendicular direction of spreading, as the process is invariant along this direction. Moreover in order to get better accuracy on the profiles, we performed a rolling average in time with a time window of 40s. This rolling average is acceptable because the spreading process is slow, which leads to a negligible evolution of the profiles over this time interval. The evolution of the density profile for $N_c = 9.63 \pm 0.80 \times 10^6$cells/ml is shown Supplementary Fig. 5a where the color codes for the time (from yellow to dark green). These profiles are spaced out by 150s. The next step is to fit these profiles with the expected profiles obtained from the theory in order to deduce the effective diffusivity. Starting from a rectangular density profile, the solution of the diffusive spreading in an infinite system (no boundary condition) is given by:

$$\rho(y,t) = \frac{\rho_0}{2}\left[\mathrm{erf}\left(\frac{y-y_0}{Q(t)}\right) - \mathrm{erf}\left(\frac{y-y_0-\Delta y}{Q(t)}\right)\right] \tag{8}$$

where $\rho_0$ is the initial density of the band, $y_0$ is the position of the center of the band, $\Delta y$ is the width of the initial rectangular band and $Q(t) = \sqrt{4Dt}$ where D is the diffusivity. The idea is then to fit, at each time step, the experimental density profile by a function of this type and then look at the evolution of the parameter $Q(t)$. If this parameter evolves has the square root of time, then the process is diffusive. As shown Supplementary Fig. 5a the fits are quite accurate. Moreover the spreading process is indeed diffusive as shown by the evolution of $Q(t)$ in Supplementary Fig. 5b for different concentrations of algae (the color codes for the concentration). By fitting $Q(t)$ with a function of the type $\sqrt{4D_{eff}t}$ we then obtained the effective diffusivity of the particles as a function of the algae concentration which is presented Figure 1c (green triangles). It is worth noticing that the initial band of colloids is not as sharp as a rectangular function. As a consequence, the initial condition of the experiments can be approximated by a profile given by $\rho(y, t_0)$ where $t_0$ would be the time needed to reach this profile from a perfect rectangular one. Then the parameter $Q(t)$ obtained experimentally has to be fitted with the function $\sqrt{4D_{eff}(t + t_0)}$, but for the sake of clarity we present in Supplementary Fig. 5b the rescaled parameter $R(t) = \sqrt{Q(t)^2 - Q(t_0)^2}$.

## SUPPLEMENTARY METHODS 3 : ANALYSIS OF JUMPS STATISTICS

Here we describe the method to extract the jumps from the recorded trajectories. This is done following 3 distinct steps. First we localize relatively big displacements that cannot be due to thermal Brownian motion. Then we determine as precise as possible where the jump starts and when it stops. This step is performed in order to accurately evaluate the duration of the jump. Finally, we discriminate between what we identified as particle entrainment (jumps) and far-field perturbation (loops) [1, 2].

To look for big (non Brownian) displacements, we compute the displacements performed by the particles on a time interval of 10 frames (0.4s), $\Delta r = r(t + 10) - r(t)$. We then locate displacements bigger than 10 pixels ($\sim 2.5\mu$m). This ensures that we probe other mechanisms than simple thermal Brownian motion. Indeed the standard deviation of the displacements due to Brownian motion is given by $\sqrt{\langle \Delta r^2 \rangle} = \sqrt{4D_0\Delta t} \sim 0.7\mu$m for $D_0 = 0.28\mu\text{m}^2.\text{s}^{-1}$ and $\Delta t = 0.4$s, which is much smaller than the threshold of $2.5\mu$m. After identifying the time at which these displacements are measured, we isolate these displacements by cutting 3.2s (80 frames) of the trajectories around this time. This step ensures that we have isolated the entire displacement. At this step the stored displacements that we have are composed of Brownian motion at the beginning and at the end and the displacement that we are looking for in the middle, see Supplementary Fig. 6a. We then need to refine this cutting by removing the Brownian motion, in order to estimate accurately the duration of the jumps. This is based on the fact that any motion due to the perturbation by CR leads to a non-zero correlation between consecutive displacements (between two frames). On the contrary, consecutive displacements are uncorrelated for standard Brownian motion. Indeed on a time scale of 0.08s ($2\Delta t$, where $\Delta t$ is the time between two frames), the displacements due to Brownian motion are independent, while the interaction between CR and the beads lasts typically longer, leading to correlated displacements.

The idea is then to look at the dot product between consecutive displacements in order to remove thermal motion from the isolated trajectories. Indeed the average dot product $\langle \vec{dr}(t + \Delta t).\vec{dr}(t) \rangle$ between consecutive displacements is 0 with a standard deviation given by $\sqrt{8}D_0\Delta t = 0.0317\mu\text{m}^2$ in the Brownian case with $D_0 = 0.28\mu\text{m}^2.\text{s}^{-1}$ and $\Delta t = 0.04$s. For each isolated trajectories, we computed the dot product $p_{\Delta t}(t) = \langle \vec{dr}(t + \Delta t).\vec{dr}(t) \rangle$ and considered the average signal $q_{\Delta t}(t) = (p_{\Delta t}(t) + p_{\Delta t}(t + \Delta t))/2$, see Supplementary Fig. 6b. This last step is intended to smooth the outcoming signal (2 consecutive displacements due to Brownian motion are still likely to be in the same direction, but this is much less probable for 3 consecutive displacements). Next we considered the binary signal $b(t)$ given by:

$$b(t) = \begin{cases} 0 & \text{if } q_{\Delta t}(t) \leq q_s \\ 1 & \text{if } q_{\Delta t}(t) > q_s \end{cases} \tag{9}$$

where the threshold $q_s = 0.0304\mu\text{m}^2$ is similar to the standard deviation of the dot product (for thermal motion). The times $t$ where the signal $b(t)$ is equal to one are assumed to correspond to directed motion. A typical signal is plotted in Supplementary Fig. 6b, which corresponds to the central trajectory in Supplementary Fig. 6a. We see that it is mainly 0 except for a quite long period of time (orange circles) and some isolated values. It is quite straightforward to recognize that the directed motion corresponds to this long period of consecutive 1 in $b(t)$, while the rest is only Brownian motion. This technique is very accurate in determining where the jumps start and stop, the error made being of the order of $2 - 3$ frames.

As shown Supplementary Fig. 6a, the first step of the protocol isolates big displacements that cannot be due to thermal motion. But this does not ensure that we have isolated only jumps (i.e. particle entrainment). Indeed when an alga swims close to a bead it will induce a loop-like motion [1, 2], which can corresponds to relatively large

displacement. An example of such motion is shown in Supplementary Fig. 6a lowest curve. In order to get statistics on the jumps only we need to discriminate between these 2 processes. To this end, we take advantage of the fact that jumps are mostly straight while loops describe curved trajectories (as the name suggests). Also jumps will in general lead to larger total displacement, even if the limit between large loops and small jumps is not very distinct. As a consequence, in order to discriminate between these two kind of mechanisms we built the following order parameter:

$$\mathcal{O} = \sqrt{\left(\sum_{i=1}^{N} \frac{dx_i}{dr_i}\right)^2 + \left(\sum_{i=1}^{N} \frac{dy_i}{dr_i}\right)^2} \cdot \Delta r, \tag{10}$$

where $dx_i = x(i+1) - x(i)$ (resp. $dy_i = y(i+1) - y(i)$) is the displacement along $x$ (resp. $y$) between two frames, $dr_i = \sqrt{dx_i^2 + dy_i^2}$, and $\Delta r$ is the total displacement. The first factor is the total displacement that the bead would have had if the length of each individual displacement had been 1. We expect this to be small for loop-like trajectories compared to more straight trajectories (jumps). The second factor is intended to increase the difference in the order parameter $\mathcal{O}$ between loops and jumps, as the total displacement again is expected to be higher for particle entrainment than for far-field perturbation. We have found that setting a threshold $\mathcal{O}_s = 10$pixels ($\sim 2.5\mu$m) on this order parameter allows us to discriminate quite accurately between the two mechanisms. But we still make some errors for small jumps, which are difficult to discriminate from loops. As a consequence the small-jumps part of the distribution $PDF(L)$ in Fig. 3a is not very accurate (for $L < L_T$). However as shown by the numerical simulations, where we only consider jumps larger than $L_T$, this part of the distribution has a negligible contribution to the dynamics.

## SUPPLEMENTARY METHODS 4 : MEASUREMENT OF THE MEAN INTERVAL TIME BETWEEN THE JUMPS

It is well known that if the total observation time of a Poisson process with parameter $\lambda$ is not sufficiently long (i.e. to be compared with the time $1/\lambda$), then the statistics of inter-arrival time is biased, even with a large ensemble average. In that case, the distribution of inter-arrival time is still seen to be exponential, but with a smaller characteristic time than the actual $1/\lambda$. Because we have not anticipated the Poisson dynamics of the entrainment events, we did not record the bead dynamics for a sufficiently long time in order to get an accurate distribution of waiting times. However, for a Poisson process, the mean waiting time $\langle \Delta T_J \rangle = 1/\lambda$ can, by definition, be estimated accurately and simply from the number of Poisson events (jumps in our case) observed on a given observation time $T_{traj}$. Indeed the estimated number of events is given simply by $E[N(T_{traj})] = \lambda T_{traj}$. So measuring the number of events for each trajectories and dividing by the length of the trajectory allowed us to measure accurately the mean interval time between the jumps: $\langle \Delta T_J \rangle = \langle T_{traj,p}/N_{J,p} \rangle_p$ where the index $p$ stands for the particles. This explains why we do observe an exponential distribution of waiting time between the jumps, but characterized by a characteristic time smaller than the actual one (see Fig. 3b and 3b-Inset), which is accurately estimated by the method explained above.

### Supplementary References

[1] Dunkel, J. Putz, V. B. Zaid, I. M. and Yeomans, J. M. Swimmer-tracer scattering at low Reynolds number. *Soft Matter* **6,** 4268-4276 (2010).
[2] Lin, Z. Thiffeault, J.-L. and Childress, S. Stirring by squirmers. *J. Fluid Mech.* **669,** 167-177 (2011).
[3] Leptos, K. C. Guasto, J. S. Gollub, J. P. Pesci, A. I. and Goldstein, R. E. Dynamics of enhanced tracer diffusion in suspensions of swimming eukaryotic microorganisms. *Phys. Rev. Lett.* **103,** 198103 (2009).
[4] Méndez, V. Campos, D. and Bartumeus, F. *Stochastic Foundations in Movement Ecology* (Springer Complexity, 2014), Ch. 3.3.
[5] Thiffeault, J.-L. *Phys. Rev. E* **92**, 023023 (2015).
